# CD11b^+^, Ly6G^+^ Cells Produce Type I Interferon and Exhibit Tissue Protective Properties Following Peripheral Virus Infection

**DOI:** 10.1371/journal.ppat.1002374

**Published:** 2011-11-10

**Authors:** Matthew A. Fischer, Michael L. Davies, Irene E. Reider, Erica L. Heipertz, Melanie R. Epler, Janet J. Sei, Molly A. Ingersoll, Nico Van Rooijen, Gwendalyn J. Randolph, Christopher C. Norbury

**Affiliations:** 1 Department of Microbiology and Immunology, Pennsylvania State University Milton S. Hershey Medical Center, Hershey, Pennsylvania, United States of America; 2 Department of Gene and Cell Medicine, Mount Sinai School of Medicine, New York, New York, United States of America; 3 Department of Molecular Cell Biology, Faculty of Medicine, Vrije Universiteit, Amsterdam, The Netherlands; Ludwig-Maximilians-Universität München, Germany

## Abstract

The goal of the innate immune system is containment of a pathogen at the site of infection prior to the initiation of an effective adaptive immune response. However, effector mechanisms must be kept in check to combat the pathogen while simultaneously limiting undesirable destruction of tissue resulting from these actions. Here we demonstrate that innate immune effector cells contain a peripheral poxvirus infection, preventing systemic spread of the virus. These innate immune effector cells are comprised primarily of CD11b^+^Ly6C^+^Ly6G^-^ monocytes that accumulate initially at the site of infection, and are then supplemented and eventually replaced by CD11b^+^Ly6C^+^Ly6G^+^ cells. The phenotype of the CD11b^+^Ly6C^+^Ly6G^+^ cells resembles neutrophils, but the infiltration of neutrophils typically occurs prior to, rather than following, accumulation of monocytes. Indeed, it appears that the CD11b^+^Ly6C^+^Ly6G^+^ cells that infiltrated the site of VACV infection in the ear are phenotypically distinct from the classical description of both neutrophils and monocyte/macrophages. We found that CD11b^+^Ly6C^+^Ly6G^+^ cells produce Type I interferons and large quantities of reactive oxygen species. We also observed that depletion of Ly6G^+^ cells results in a dramatic increase in tissue damage at the site of infection. Tissue damage is also increased in the absence of reactive oxygen species, although reactive oxygen species are typically thought to be damaging to tissue rather than protective. These data indicate the existence of a specialized population of CD11b^+^Ly6C^+^Ly6G^+^ cells that infiltrates a site of virus infection late and protects the infected tissue from immune-mediated damage via production of reactive oxygen species. Regulation of the action of this population of cells may provide an intervention to prevent innate immune-mediated tissue destruction.

## Author Summary

During a natural virus infection, small doses of infectious virus are deposited at a peripheral infection site, and then a “race” ensues, in which the replicating virus attempts to “outpace” the responding immune system of the host. In the early phases of infection, the innate immune system must contain the infection prior to the development of an effective adaptive response. Here we have characterized the cells of the innate immune system that move to a site of peripheral virus infection, and we find that a subset of these cells display atypical expression of cell surface molecules, timing of infiltration, and function. These cells protect the infected tissue from damage by producing reactive oxygen molecules, which are widely accepted to increase tissue damage. Therefore our findings indicate that during a peripheral virus infection, the typical rules governing the function of the innate immune system are altered to prevent tissue damage.

## Introduction

Typically, the acute innate immune response to a peripheral challenge involves rapid infiltration of Ly6C^+^Ly6G^+^ neutrophils, followed by Ly6C^+^Ly6G^-^ monocytes, in a process that involves chemoattraction mediated by arachidonic acid metabolites, cytokines, and chemokines [1]. Both neutrophils and monocytes mediate inflammation, but monocytes are also thought to play a major role in clearance of apoptotic neutrophils and restoration of tissue homeostasis [2], [3]. Neutrophils and monocytes are not, however, homogeneous populations of cells, and subtypes of these cells have been described based on their expression of surface markers or production of cytokines. A full understanding of the phenotype and function of each of these cell populations is required in order to understand (and manipulate) the mechanisms that clear pathogens, prevent systemic spread, and prevent or reduce immune–mediated tissue damage at the site of infection.

The majority of studies investigating the role of innate immune effector cells have been conducted using either sterile inflammation models or bacterial infections. Here we have examined the role of innate immune effector cells in protection against peripheral infection with virus. Many investigations studying antiviral immunity have utilized systemic routes of infection (intraperitoneal or intravenous) or examined infections in the respiratory tract. However, numerous viral infections are transmitted through breaks in the skin, and the dermal route of inoculation is favored for delivery of viral vaccine vectors [4], [5], [6]. Following infection of the skin with a pathogenic virus, replication occurs locally unless controlled by the innate immune system, and subsequently the virus spreads systemically to cause disease. After intradermal infection with vaccinia virus (VACV), a natural peripheral route of infection [7], the immune system prevents systemic spread of the virus [8]. A large number of the infiltrating cells at the site of infection are F4/80^+^, likely representing monocytes/macrophages [9], [10]. Although CD4^+^ T cells and antibodies have been implicated in the control of VACV infection following systemic challenge [11], the cells responsible for preventing systemic spread of VACV following an intradermal infection have not been identified.

Several recent studies have described important roles for monocytic cells in the immune responses to various intracellular pathogens [12], [13], including viruses [14], [15], [16]. In respiratory infection, depletion of alveolar macrophages enhances the spread of VACV to peripheral sites such as the ovaries, indicating that these cells may play an important role in anti-VACV immunity [17]. Following systemic infection with VACV, TLR2-mediated recognition of uncharacterized viral components causes both IL-6 production [18] and Type I interferon production by Ly6C^+^CD11b^+^ cells [19], resulting in a reduction in virus titers. However, the role of these innate immune effector cells and molecules in control of virus spread from a natural peripheral site of infection as well as their role in tissue regeneration following infection, has not been addressed. In addition, the role of Ly6C^+^Ly6G^+^ cells in protective immunity and tissue protective responses following VACV infection is unknown.

Here we describe the role of Ly6C^+^Ly6G^-^ monocytes in preventing systemic spread of virus from a dermal site of infection without a requirement for T cell infiltration. In addition, we describe the role of Ly6C^+^Ly6G^+^ cells that infiltrate the site of infection subsequent to the accumulation of monocytes. These Ly6C^+^Ly6G^+^ cells produced Type I interferon and mediated tissue repair via the production of reactive oxygen species. These findings demonstrate the complexity of the cellular innate response to peripheral virus infections, and how the response differs from innate responses to a sterile inflammatory stimulus. Thus, our results demonstrate the plasticity of innate immune effector compartments, describe a previously unknown role for a subset of Ly6C^+^Ly6G^+^ cells, and show that reactive oxygen species (ROS) production by these cells allows the resolution, rather than the exacerbation, of tissue damage during an acute infection.

## Results

### Phagocytes prevent the systemic spread of VACV following peripheral infection

**Figure 1:**
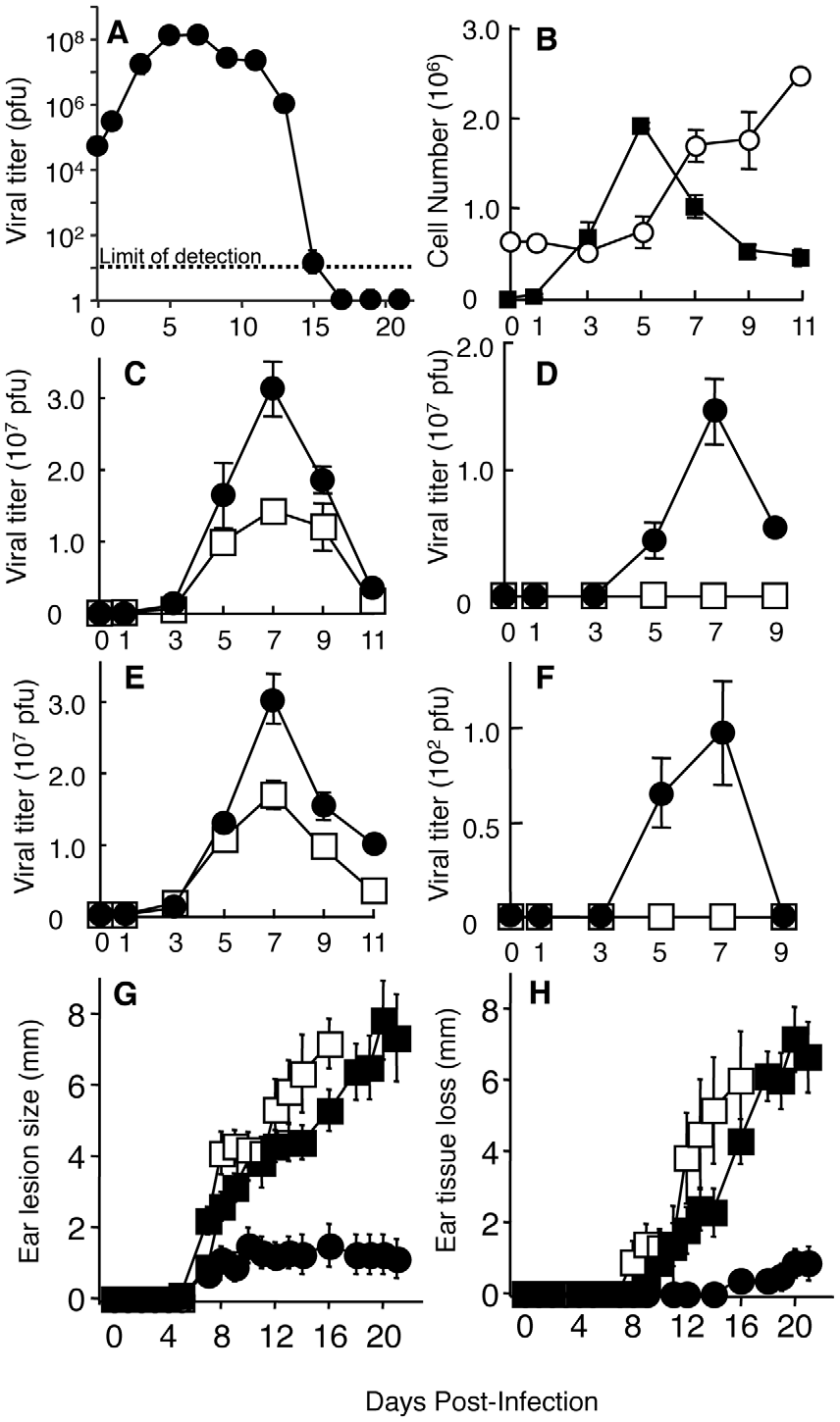
Phagocytic cells control the systemic spread of virus from the ear pinnae and tissue damage at the site of infection. Wild-type (A–D, G, H) or MAFIA (E, F, G, H) mice were infected i.d. in the ear pinnae with VACV. Ear pinnae or ovaries were harvested at various days post-infection. Ear (A, C, E) or ovary (D, F) lysates were used in a plaque assay to determine viral replication kinetics *in vivo*. B) Immune cells were isolated from infected ear pinnae and identified as either T cells (open circles; TCRβ^+^, CD90^+^) or phagocytes (filled squares; CD11b^+^, CD90^-^, NK1.1^-^, CD19^-^) using flow cytometry. Infected ear pinnae (C, E) and ovaries (D, F) were harvested from clodronate liposome-(C, D; filled circles) or vehicle-(C, D; open squares) treated wild-type mice and AP20187-(E, F; filled circles) or vehicle-(E, F; open squares) treated MAFIA mice. G, H) The tissue damage in wild-type mice treated with clodronate liposomes (filled squares) or vehicle (filled circles), or MAFIA mice treated with AP20187 (open squares) was documented on indicated days post infection, by measuring lesion size (G) or loss of tissue (H). Data is representative of at least three independent experiments and data shown represent mean cell numbers or virus titers +/- SEM from four ears at each time point and mean lesion size or tissue damage +/- SEM from 10 ears mice at each time point are depicted in (G, H).

To gain insight into the mechanisms deployed by the immune system to prevent the systemic spread of virus following a peripheral infection, we infected mice in the ear with VACV and monitored both virus replication and the infiltration of populations of immune effector cells to the ear at various times post infection. Virus replicated exponentially in the ear pinnae until day 5, at which point the titer began to plateau (**Fig. 1A**). Virus titers then dropped until virus was finally cleared when the scab that formed at the site of infection fell off between day 12 and 15 post infection. At day 5 post infection, the time point at which virus replication plateaus, no significant accumulation of αβ TCR T cells was observed at the site of infection (**Fig. 1B**). However, the plateau of virus replication coincided with the peak in numbers of CD11b^+^ cells (**Fig. 1B**). The CD11b^+^ population contained a small but reproducible number of CD11c^lo^ cells that expressed CD11b, but not B220 (CD45RA), and likely represent monocyte-derived DC (**Fig. S1**). We did not observe infiltration of CD11c^+^ B220^+^ plasmacytoid DC, proposed to be the primary producer of Type I IFN, to the site of infection up to day 11 post infection (data not shown).

To determine the role of the infiltrating CD11b^+^ cells, we intravenously injected clodronate liposomes to induce apoptosis of the phagocytic cells that internalize the liposomes [20]. We typically observed a 70–80% depletion of CD11b^+^ cells at the site of infection throughout the time course of the experiment upon repeated injection of clodronate liposomes (data not shown). Liposome injection resulted in a minor increase in VACV titers in the ear pinnae of the phagocyte-depleted mice on days 5–9 as compared to control mice (**Fig. 1C**). VACV replication is completely confined to the ear pinnae following intradermal infection with a dose of 10^4^ pfu [8]. The confinement of virus replication to the ear pinnae contrasts with systemic routes of infection, such as intraperitoneally or intravenously, after which virus can be found in multiple organs and tissues, including the ovaries, the primary site of VACV replication in a female mouse. Because the spread of VACV to the ovaries is accelerated following the depletion of alveolar macrophages after an intranasal challenge [17], we examined the role of phagocytes in the confinement of viral replication to the ear pinnae. The presence of replicative VACV in the ovaries was analyzed using a plaque assay following depletion with clodronate liposomes. Replicative VACV was observed in the ovaries of mice undergoing clodronate liposome treatment, but not in control mice (**Fig. 1D**). VACV was detected in the ovaries beginning on day 5 and peaked on day 7 at a level 1000-fold in excess of the original inoculum. Notably, depletion of T cells systemically and in the ear using an anti-Thy1 antibody did not increase virus titers in the ear through day 9 post infection (**Fig. S2**) or allow detection of VACV in the ovaries (data not shown). Therefore phagocytes, but not T cells, play a vital role in controlling virus replication at the site of infection and in preventing the systemic spread of VACV following a peripheral infection.

CD11b is a broadly expressed integrin subunit that is found on neutrophils, monocytes, macrophages and some DC subsets. In an attempt to specifically address the role of monocyte/macrophages in control of virus replication at the site of infection and the spread of VACV systemically, we utilized Macrophage Fas-Induced Apoptosis (MAFIA) mice which encode both a suicide gene and Green Fluorescent Protein (GFP) driven by the *c-fms* (CD115/MCSF receptor) promoter [21]. Injection of the drug AP20187 leads to dimerization of the suicide protein, activation of the Fas pathway, and subsequent apoptosis of cells expressing CD115. Previous publications have shown that monocyte/macrophages in MAFIA mice are GFP^+^ and are depleted upon AP20187 treatment, whereas neutrophils express very little GFP and are unaffected by AP20187 treatment in these mice [21]. In our studies, repeated injections of AP20187 produced >80% depletion of CD11b^+^ cells at the site of infection (data not shown). Similar to clodronate liposome treatment, AP20187 treatment of MAFIA mice produced a minor but reproducible increase in VACV titers in the ear of infected mice (**Fig. 1E**). AP20187 administration to MAFIA mice also allowed systemic spread of virus in the majority of mice (**Fig. 1F**), but the VACV titers found in the ovaries of treated MAFIA mice were 10^5^-fold lower than titers seen following clodronate liposome treatment (**Fig. 1D**). These data indicated that the primary cell type required for preventing systemic spread of VACV is a subset of phagocytic cells that was depleted effectively in mice treated with clodronate liposomes, but less effectively depleted in MAFIA mice treated with AP20187.

VACV infection in the ear causes significant pathology that can be quantified by measurement of swelling, lesion size and tissue damage [8]. The pathology we observed following VACV infection differed slightly from published reports, a finding that is likely caused by differing sources of laboratory animals and different housing conditions between institutions. When we depleted mice of phagocytes using either clodronate or AP20187 treatment, the pathology at the site of VACV infection was dramatically increased at late times. To quantify this pathology, we measured the lesion size and tissue damage, which represents the loss of necrotic tissue from the ear [8], in infected mice that were untreated or depleted using clodronate liposomes or AP20187 . Both lesion size and tissue loss was significantly greater in mice depleted of phagocytes using either methodology (**Fig. 1G, H**). Notably, both the increase in lesion size and the tissue loss in phagocyte-depleted mice occurred primarily at time points after the minor increase in virus replication had been controlled. These data indicate a dual role for phagocytes during VACV infection, namely blockade of systemic spread and reduction of host-mediated tissue pathology.

### Distinct monocyte populations migrate to the site of VACV infection

**Figure 2:**
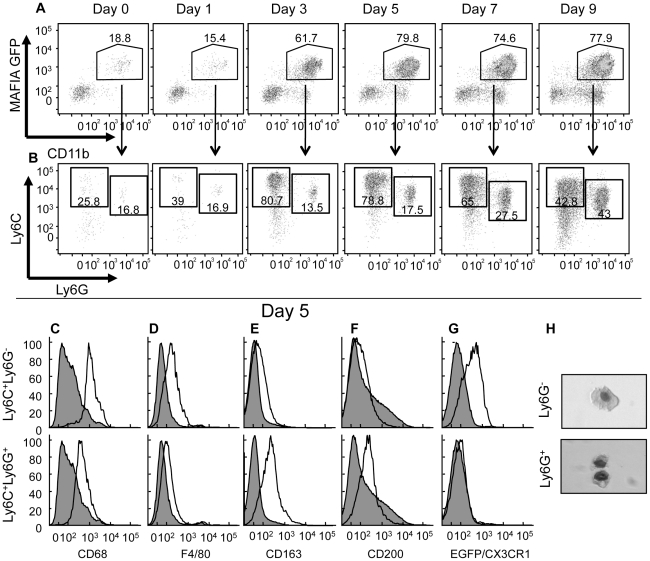
Ly6C^**+**^Ly6G^-^ and Ly6C^**+**^Ly6G^**+**^ subpopulations of cells at the site of VACV infection. MAFIA (A–F), CX3CR1^+/GFP^ (G) or wild-type (H) mice were infected in the ear pinnae with VACV and the ears harvested at various days post infection (A, B) or on day 5 post infection (C–H). A) The phagocyte population (CD11b^+^, CD90^-^, NK1.1^-^, CD19^-^) in the ear was analyzed (using a gating strategy outlined in Fig. S3) for expression of GFP (driven by the CD115 promoter) and CD11b. B) The GFP^+^CD11b^+^ population from A) was analyzed for expression of Ly6C and Ly6G. C–G) The resulting Ly6C^+^Ly6G^-^ (open histograms, top row) and Ly6C^+^Ly6G^+^ (open histograms, bottom row) populations were analyzed for the expression of various intracellular (C) or cell surface (D–F) markers using antibodies or GFP as a surrogate for CX3CR1 expression (G) and compared to CD11b^-^ cells (filled histogram). H) Cells from ear pinnae 5 days post-infection were magnetically sorted based on Ly6G expression, fixed onto slides using cytofugation, and stained. Data shown are representative of at least three independent experiments in which three mice were examined at each time point.

In order to investigate which cell population was required to prevent spread of VACV from the site of infection, we needed to further characterize the phenotypes of cells within the infiltrating CD11b^+^ population at the site of infection. We examined infiltration of CD11b^+^ cells to the ear of MAFIA mice at various times post infection, taking care to ensure that the cells that we observed were not doublets (which could confound their identity) as outlined in our gating strategy (**Fig. S3**). Virtually all of the CD11b^+^ cells infiltrating the site of infection were GFP^+^ in MAFIA mice (**Fig. 2A**), until day 7 post infection when a CD11b^+^ GFP^-^ population began to accumulate [9]. Thus, either all CD11b^+^ cells infiltrating the site of infection were of monocytic origin or the use of a VACV infection model led to expression of GFP within cells in MAFIA mice that were not of monocytic origin. Therefore it was necessary to distinguish monocyte/macrophages from neutrophils, which comprise another likely major population of infiltrating phagocytes.

There are few phenotypic markers that can distinguish neutrophil and monocytes/macrophage populations. One of these markers is CD115, but we were unable to detect infiltrating CD11b^+^GFP^+^CD115^+^ cells at the site of VACV infection (data not shown) although similar digestion protocols in different tissues in uninfected mice did produce CD115 staining. As surrogate GFP expression driven by the CD115 promoter did not distinguish between these cells types (**Fig. 2A**), we examined expression of cell surface Ly6C and Ly6G, as published work indicates that Ly6C is expressed by monocytes [22], [23], whereas both Ly6C and Ly6G are expressed by granulocytes [24], [25]. Using antibodies specific for Ly6C and Ly6G to analyze the CD11b^+^ cell infiltrate by flow cytometry, we observed several different cell populations accumulating in the ear over the course of the infection (**Fig. 2B**). The accumulation of immune cell populations, including Ly6C^+^Ly6G^+^ neutrophils, did not occur until day 2–3 post infection (**Fig. 2A–B**).

A population of Ly6C^+^Ly6G^-^ monocytes was detected in the ear at numbers above uninfected tissue on day 3 post infection and represented the predominant CD11b^+^ cell at the site of infection through day 7 (**Fig. 2B**). Small numbers of Ly6C^+^Ly6G^+^ cells could be identified in the ear at early times after infection, but the numbers of these cells increased significantly after 5 days of infection and became the major population beyond day 7. Typically, this Ly6C^+^Ly6G^+^ population would be classified as neutrophils. However, the timing of infiltration of these Ly6C^+^Ly6G^+^ cells is not consistent with the infiltration of neutrophils that infiltrate a site of insult early, before monocytes, and die rapidly unless replaced [2]. Similar populations of infiltrating Ly6C^+^Ly6G^+^ cells were observed at the site of infection 5 days after dermal infection with other viruses (**Fig. S4**), indicating that the later infiltration of Ly6C^+^Ly6G^+^ cells is not specific to VACV infection. Closer analysis revealed that the Ly6C^+^Ly6G^+^ cells expressed several proteins considered to be monocyte/macrophage markers, including CD68, F4/80, CD200 and the scavenger receptor CD163 (**Fig. 2C–F**).

To investigate whether the Ly6C^+^Ly6G^+^ cells that we observed were monocytes, we infected CX_3_CR1^+/GFP^ mice in which one of the copies of the CX_3_CR1 chemokine receptor has been replaced by GFP [26]. All monocytes in these mice express GFP at high levels [26]. Following VACV infection, the CD11b^+^Ly6C^+^Ly6G^-^ cells in the ear pinnae expressed GFP (**Fig. 2G**). However, the CD11b^+^Ly6C^+^Ly6G^+^ cells did not express GFP driven by the CX_3_CR1 promoter, indicating that they are unlikely to be derived from the monocyte population (**Fig. 2G**). To further investigate the phenotype of infiltrating cell populations from the ear of VACV infected mice, we isolated Ly6G^+^ and Ly6G^-^ cells from ear pinnae 5 days post-infection by magnetic separation and visualized their nuclear morphology by microscopy. Both Ly6G^-^ and Ly6G^+^ fractions were comprised primarily of mononuclear cells (**Fig. 2H**). Therefore it appears that the Ly6C^+^Ly6G^+^ cells that infiltrated the site of VACV infection in the ear are phenotypically distinct from the classical description of both neutrophils and monocyte/macrophages.

**Figure 3:**
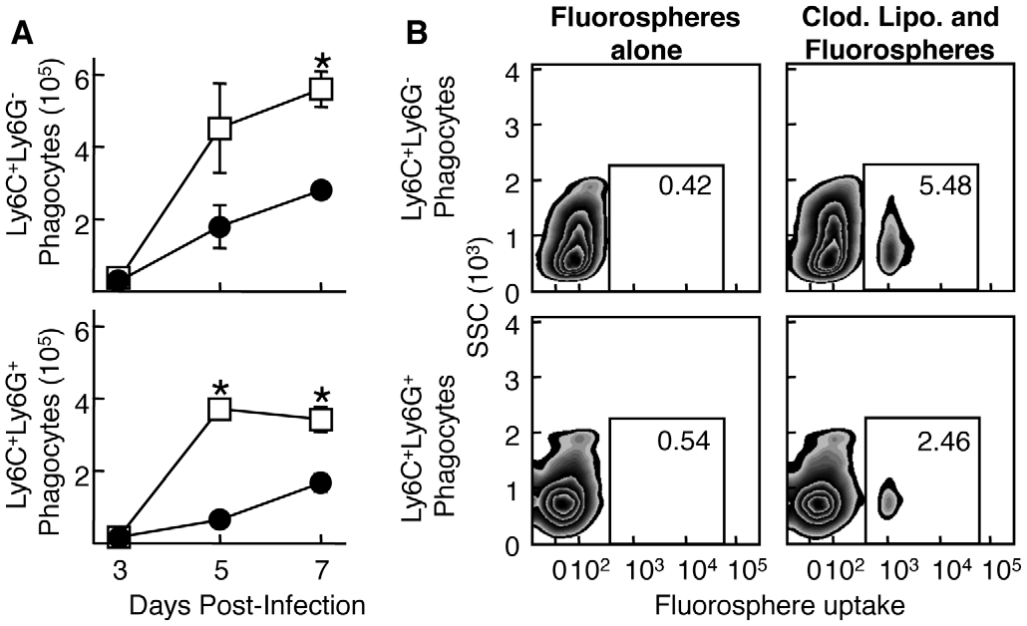
Both Ly6C^**+**^Ly6G^-^ and Ly6C^**+**^Ly6G^**+**^ subpopulations extravasate into the site of infection. A) Mice were treated i.v. with either non-depleting anti-CD11b (filled circles) or isotype control (open squares) and infected i.d. in the ear pinnae with VACV. Ear pinnae were harvested at various times post-infection and the phagocyte population was then analyzed for the Ly6C^+^Ly6G^-^ (top) and Ly6C^+^Ly6G^+^ (bottom) subpopulations. B) Mice either untreated (left) or treated once (as opposed to the repeated treatment in Fig. 1) with clodronate liposomes (right) were injected with fluorospheres and then infected i.d. with WR VACV. After 5 days of infection, ear pinnae were harvested and Ly6C^+^Ly6G^-^ (top) and Ly6C^+^Ly6G^+^ (bottom) phagocytes were analyzed for fluorosphere uptake using flow cytometry. Data are representative of at least three independent experiments and mean phagocyte numbers +/- SEM from four ears at each time point are depicted in A).* represents p<0.05 using a Student's T-test.

To identify whether Ly6C^+^Ly6G^+^ cells migrate to the site of infection or expand *in situ,* we blocked infiltration of these cells to the infection site with a non-depleting antibody targeting the integrin subunit CD11b. Anti-CD11b antibody treatment reduced infiltration of both Ly6C^+^Ly6G^-^ and Ly6C^+^Ly6G^+^ cells equally (**Fig. 3A**), indicating a similar requirement for transport across the endothelial cell layer for each of these cell populations. To study whether the Ly6C^+^Ly6G^+^ cell population we observed at the site of VACV infection is derived from the circulation, we injected fluorescent fluorospheres i.v. and examined the migration of cells that had ingested the fluorospheres to the site of VACV infection. Prior to fluorosphere injection, we depleted circulating monocytes with clodronate to ensure that we labeled cells that were mobilized from the bone marrow [27]. Similar profiles of Ly6C^+^Ly6G^+^ and Ly6C^+^Ly6G^-^ cells in the ear contained fluorospheres after internalization in the blood (**Fig. 3B**), suggesting that both populations repopulate the circulation following clodronate depletion of monocytes. Taken together, these data indicate that neither population of cells is likely to be derived from resident cells, but rather each moves into the site of infection from the blood.

### Function of the monocyte/macrophage subpopulations

**Figure 4:**
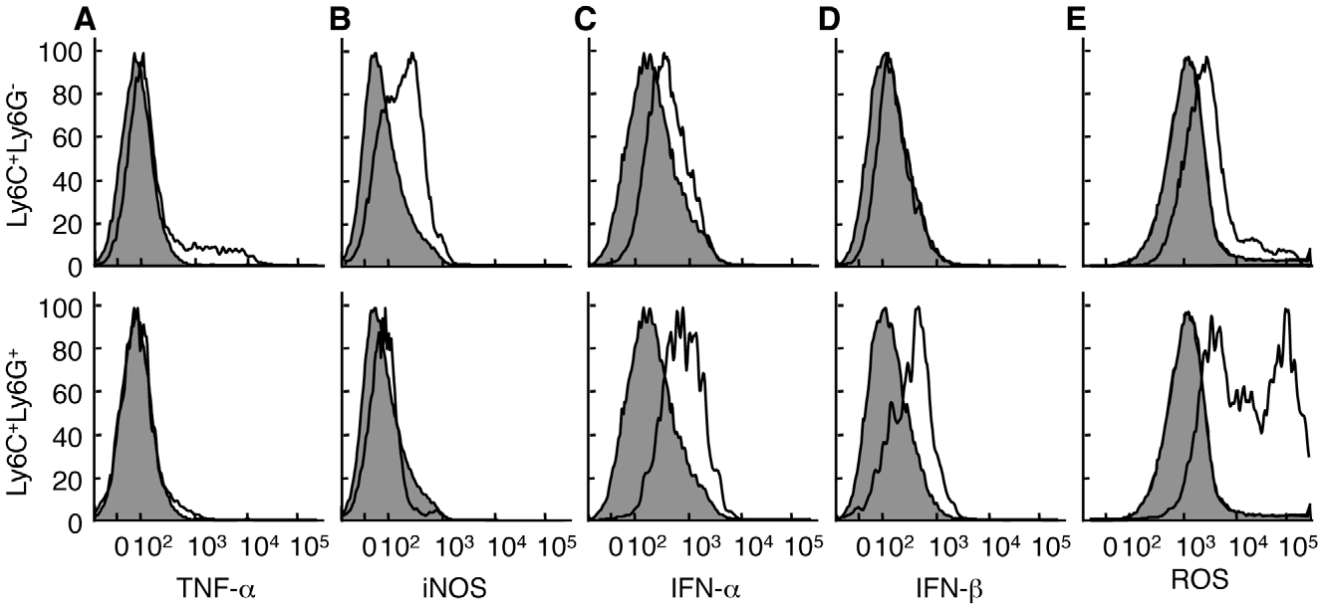
Ly6C^**+**^Ly6G^-^ and Ly6C^**+**^Ly6G^**+**^ cells have distinct functional profiles. Mice were infected i.d. in the ear pinnae with VACV. Ear pinnae were harvested at 5 days post-infection. The Ly6C^+^Ly6G^-^ (top, open histograms) and Ly6C^+^Ly6G^+^ (bottom, open histograms) CD11b^+^ monocyte subpopulations were analyzed for the expression of various cytokines/effector molecules using antibodies (A-D) or a reactive dye triggered by exposure to ROS (E) and compared to CD11b^-^ cells (filled histograms). For TNF-α staining, cells were first incubated with CpG DNA. Data are representative of at least three independent experiments in which the phenotype of at least two mice were examined independently.

The true measure of cellular specialization is best demonstrated by the dedicated function of a cell type. Thus, we analyzed the Ly6C^+^Ly6G^+^ and Ly6C^+^Ly6G^-^ populations for differences in function. The Ly6C^+^Ly6G^-^ population at the site of infection expressed inducible nitric oxide synthase (iNOS) and, when exposed to CpG oligonucleotides, a portion of these Ly6C^+^Ly6G^-^ cells produced TNF-α (**Fig. 4A, B**). In contrast, none of the Ly6C^+^Ly6G^+^ cells expressed iNOS above the level of CD11b^-^ cells, nor did the Ly6C^+^Ly6G^+^ cells produce TNF-α either directly *ex vivo* or following stimulation with CpG oligonucleotides. Although both TNF-α [28] and iNOS [29], [30] have been reported to be required for efficient control of VACV replication in mice, we did not observe any difference in VACV replication in mice lacking iNOS compared to wild-type mice (**Fig. S5**). Production of Type I interferons is also essential to control VACV replication *in vivo* [31], [32] so we examined production of IFN-α and IFN-β by Ly6C^+^Ly6G^-^ and Ly6C^+^Ly6G^+^ cells at the site of VACV infection. We found that Ly6C^+^Ly6G^+^ cells produced significant levels of Type I IFN when compared to either CD11b^-^ cells or Ly6C^+^Ly6G^-^ cells when directly isolated from the site of VACV infection (**Fig. 4C, D**). Notably, this production of Type I IFN occurred without the need for any additional stimulation. Ly6C^+^Ly6G^+^ neutrophils typically produce large quantities of ROS, so we incubated cells isolated from VACV-infected ears with a dye that becomes fluorescent upon exposure to ROS (CM-H_2_DCFDA). Ly6C^+^Ly6G^-^ cells stained with the ROS substrate at a higher level than CD11b^-^ cells, but Ly6C^+^Ly6G^+^ cells produced much higher levels of ROS (up to a 2 log_10_ shift in fluorescence) without additional stimulation (**Fig. 4E**). These data indicate that the Ly6C^+^Ly6G^-^ and Ly6C^+^Ly6G^+^ cells are functionally distinct, and demonstrate that both cell types provide functions important in the control of VACV replication.

### Ly6G^+^ cells are tissue protective

**Figure 5:**
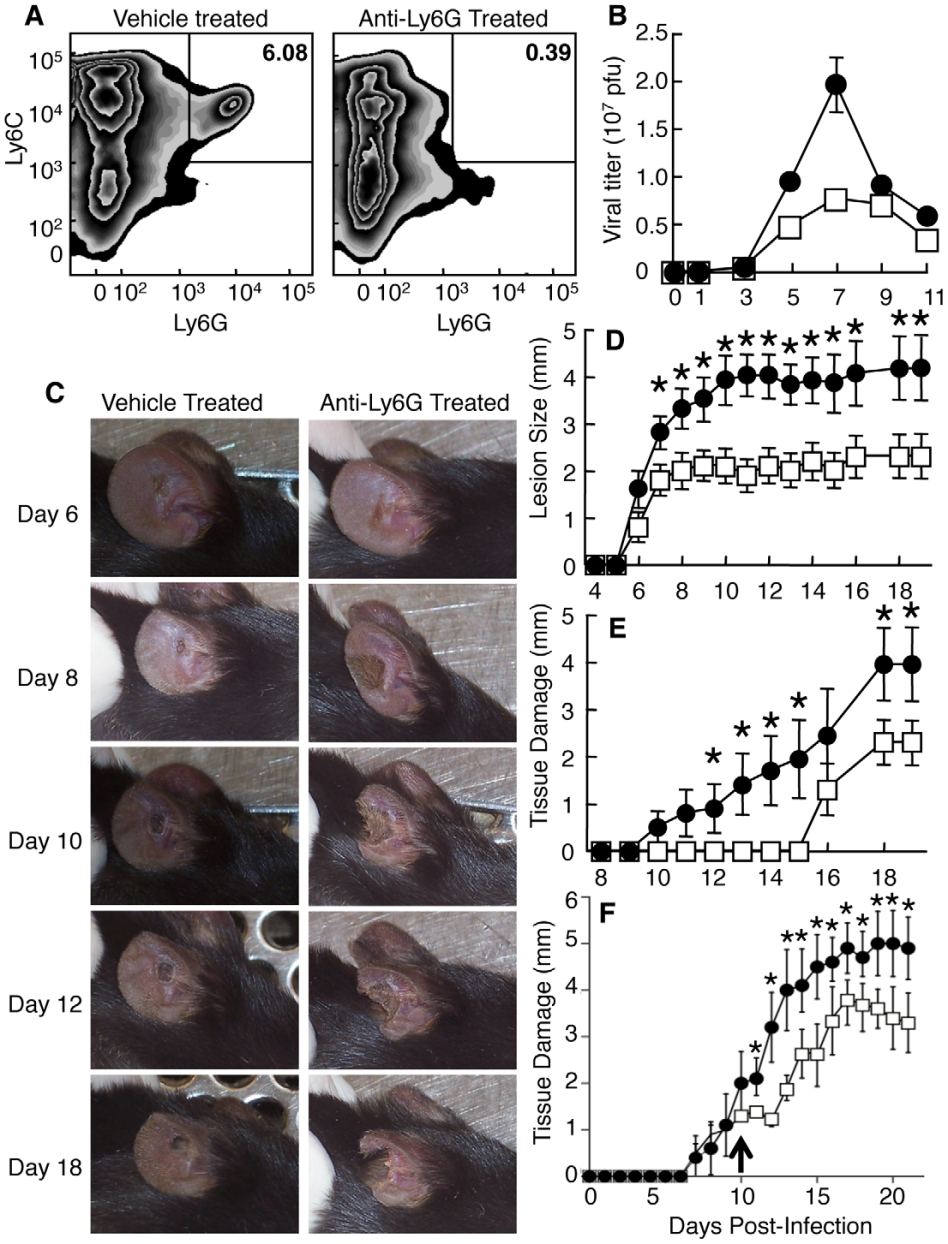
Depletion of Ly6G^**+**^ cells leads to increased tissue damage at the site of VACV infection. Mice were treated with vehicle (A-left, B, D, E - open squares), isotype control antibody (F-open squares) or anti-Ly6G antibody (A-right, B, D, E, F - filled circles) and infected i.d. in the ear pinnae with VACV. A) The presence of Ly6C^+^Ly6G^-^ and Ly6C^+^Ly6G^+^ monocytes populations in the ear pinnae was measured 5 days post-infection. B) Replicating virus titers were measured at various times post infection. C-F) The tissue damage in mice treated with anti-Ly6G antibody for the entire course of infection (C, D, E) or after day 10 post infection (F, arrowed) was documented visually on indicated days post infection (C), by measuring lesion size (D) or loss of tissue (E, F). Data are representative of at least three independent experiments. Mean virus titers +/- SEM from four ears at each time point are depicted in (B). Mean lesion size or tissue damage +/- SEM from five mice at each time point are depicted in (D, E). * represents p<0.05 using a Student's T-test.

To ascertain whether production of Type I IFN by Ly6C^+^Ly6G^+^ cells is required for efficient control of VACV replication at the site of infection, we depleted Ly6G^+^ cells using 1A8 antibody specific for Ly6G. This antibody depletes Ly6G^+^ cells while leaving Ly6C^+^Ly6G^-^ cells unaffected, in contrast to antibodies used to deplete Gr1^+^ cells, such as RB6-8C5 [25] (**Fig. 5A**). Mice receiving the anti-Ly6G 1A8 antibody displayed a modest 2.5-fold increase in virus replication in the ear pinnae on days 5 and 7 post-infection (**Fig. 5B**) comparable to that observed following clodronate liposome treatment or MAFIA-dependent depletion. In contrast to treatments that globally deplete monocyte/macrophages, such as clodronate treatment, administration of the 1A8 antibody resulted in undetectable levels of replicative VACV in the ovaries (data not shown). Virus replication in the ear was controlled by day 9 post-infection. Therefore, Ly6G^+^ cells, including the population of Ly6C^+^Ly6G^+^ cells we observed at the site of infection, likely play only a minor role in reducing virus replication and spread.

When we depleted mice of Ly6G^+^ cells, the pathology at the site of VACV infection was dramatically increased and large areas of infected ears became necrotic and eventually fell off (**Fig. 5C**). To quantify this pathology, we measured the lesion size and tissue damage, which represents the loss of necrotic tissue from the ear [8], in infected mice that were vehicle treated or depleted of Ly6G^+^ cells. Both lesion size and tissue loss was significantly greater in mice depleted of Ly6G^+^ cells (**Fig. 5D, E**) than in those treated with isotype control antibody (**Fig. S6**). Notably, both the increase in lesion size and the tissue loss in anti-Ly6G antibody-treated mice occurred primarily at time points after the minor increase in virus replication had been controlled. To ensure that the tissue protective function of Ly6G^+^ cells occurred after the control of VACV replication we injected anti-Ly6G antibody or isotype control on day 10 post-infection and monitored lesion size (not shown) and tissue damage (**Fig. 5F**). As above, depletion of Ly6G^+^ cells following control of virus replication enhanced tissue damage. These data indicate a tissue protective role for Ly6G^+^ cells during VACV infection.

### Tissue protection is mediated by ROS

**Figure 6:**
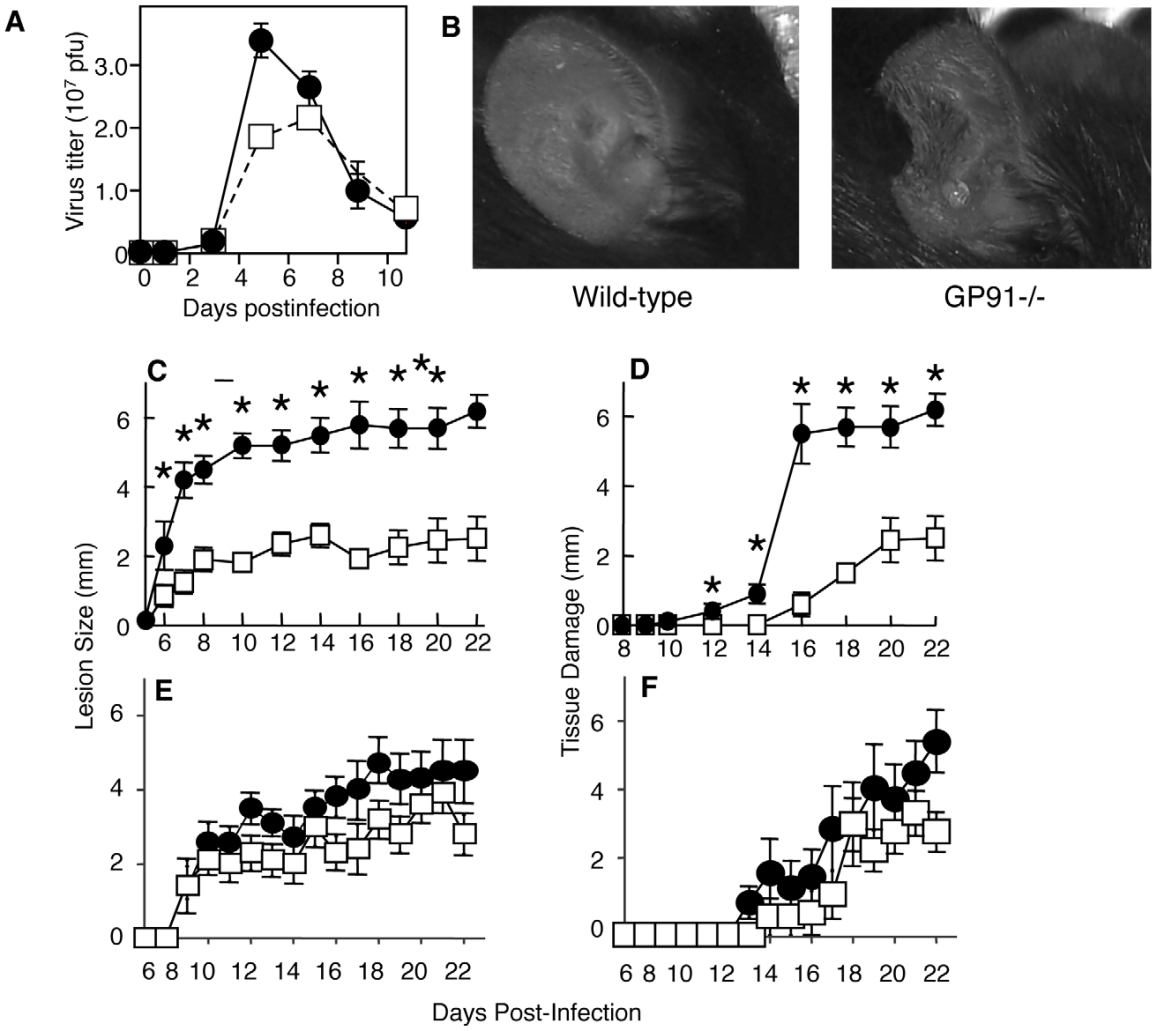
Loss of NADPH-oxidase activity leads to increased tissue damage at the site of VACV infection. A–D) Wild-type (open squares) or GP91^-/-^ (filled circles) mice were infected i.d. in the ear pinnae with VACV. A) Replicating virus titers in the ear pinnae were measured at various times post infection. The tissue damage in GP91^-/-^ mice was documented visually on day 9 (B), or by measuring lesion size (C) or loss of tissue (D) at various times post infection. E, F) GP91^-/-^ mice were treated with anti-Thy1 (closed circles) or isotype control (open squares) and lesion size (E) and tissue damage (F) quantified at the days shown. Mean virus titers +/- SEM from four ears mice at each time point are depicted in (A). Mean lesion size or tissue damage +/- SEM from ten ears at each time point are depicted in (C, D, E F). * represents p<0.05 using a Student's T-test.

Because production of ROS is the major functional phenotype of Ly6C^+^Ly6G^+^ cells following VACV infection, we investigated the role of ROS production in control of virus replication in the ear, control of virus spread to the ovaries, and tissue protection. We infected gp91^-/-^ mice that lack the membrane component of the phagocyte NADPH oxidase, and therefore cannot generate ROS [33]. Gp91^-/-^ mice displayed slightly (0.5-fold) enhanced replication of VACV in the ear at day 5 post-infection (**Fig. 6A**) and, similar to wild-type mice, no replicating virus could be detected in the ovaries (data not shown). However, the ears of gp91^-/-^ mice displayed very similar characteristics upon infection to those of mice depleted of Ly6G^+^ cells, namely that large portions of the ear became necrotic and were eventually shed (**Fig. 6B**). When we quantified lesion size and tissue loss as outlined above, we observed that a lack of ROS significantly increased tissue damage at time points when there was no effect upon virus replication (**Fig. 6C-D**). There was no significant difference between the infiltration of CD11b^+^ or Ly6C^+^Ly6G^+^ cells between wild-type and gp91^-/-^ mice, indicating that a difference in chemotaxis of tissue protective cells did not account for the difference in tissue damage observed in gp91^-/-^ mice (**Fig. S7**). In addition, depletion of Ly6G+ cells in gp91^-/-^ mice did not exacerbate or ameliorate tissue damage (**Fig. S8**) in contrast to wild-type mice, where treatment with anti-Ly6G dramatically increased tissue damage (**Fig. 5 D, E**). These data demonstrate that production of ROS, likely by Ly6C^+^Ly6G^+^ cells, prevents tissue damage following VACV infection.

The mechanisms responsible for the pathology observed at the site of VACV infection remain unknown, but a great deal of recent work has focused on the ability of myeloid cell population to suppress T cell activity. Therefore we examine the ability of T cells to induce tissue damage in mice lacking ROS. We depleted T cells with anti-Thy1 antibody (**Fig. S2**) and measured lesion size and tissue damage as above. If T cells were responsible for tissue damage and their function was modulated by ROS we would expect to observe a reduction in the tissue damage in gp91^-/-^ depleted of T cells. We did not observe a decrease in lesion size (**Fig. 6E**) or tissue damage (**Fig. 6F**) in mice treated with anti-Thy1 antibody, and in some, but not all, experiments we observed an increase in damage in T cell depleted mice. Therefore ROS-mediated modulation of tissue damage is not achieved via an effect on T cell activity.

## Discussion

In this study, we describe two populations of innate immune effectors, identified as CD11b^+^Ly6C^+^Ly6G^-^ and CD11b^+^Ly6C^+^Ly6G^+^ that migrate to a peripheral site of virus infection. These populations are phenotypically distinct, and mediate multiple functions that control virus replication, prevent systemic spread of virus, and simultaneously reduce tissue damage. We demonstrate the recruitment of a non-typical Ly6C^+^Ly6G^+^ population that appears to mediate both effector (Type I interferon production) and immunomodulatory (reduction of tissue damage) functions following virus infection. Depletion of this population of cells reveals that their function is vital in protection of tissue from catastrophic damage mediated by the inflammatory response. Manipulation of their function may allow the generation of tissue protective responses during infection to prevent immune-mediated pathology.

Our observation that Ly6C^+^Ly6G^+^ cells produce Type I interferons is in contrast to previous publications in which production of antiviral Type I IFN by innate immune effectors is typically held to be the role of plasmacytoid DC [34]. We did not observe CD11c^+^B220^+^ plasmacytoid DC at the site of infection, and we were unable to attribute an effector function such as cytokine or other inflammatory mediator production to the small number of CD11c^+^ cells with the phenotype of monocyte-derived DC at the site of infection. These “inflammatory DC” may play a role in antigen presentation to CD4^+^ T cells that migrate to the site of infection at later time points [35]. Following systemic VACV infection, TLR2-mediated recognition by CD11b^+^Ly6C^+^Ly6G^-^, but not Ly6C^+^Ly6G^+^, cells leads to the production of Type I IFN [19]. This apparent discrepancy could be explained by the ability of systemically administered VACV to reach lymphoid resident macrophage populations that have a differential ability to produce Type I interferon. The natural route of infection with VACV appears to be via touch [36], and dermal infection reveals a role for immune evasion molecules that other routes of infection do not [7]. If natural infection is via the dermal route, then only cells migrating to the site of infection may be exposed to the virus, explaining the difference in cell type producing Type I interferons in our study.

It is clear from our data that Ly6C^+^Ly6G^+^ cells are required to modulate the immune response and reduce tissue damage following infection with VACV. The enhanced damage observed following depletion of Ly6G^+^ cells is unlikely to result from the minor increases in VACV titers in the ear. A two-fold increase in virus titer is minor, as each infected cell will produce 10^2^–10^3^ progeny virions, so major changes in control of virus replication would likely produce log_10_ changes in titer. We show that Ly6C^+^Ly6G^+^ cells are present after clearance of virus, presumably to aid in recovery of the tissue from immune-mediated pathology. Modulation of tissue damage requires the production of ROS, which are often associated with tissue damage. Oxygenation is often required for tissue repair, however, and the presence of oxygen may allow greater production of oxygen radical that are required for tissue repair, or modulation of the immune response to reduce tissue damage [37], [38]. The production of ROS can modulate both T cell responses and innate immune responses [39], [40], [41], [42]. Our data indicate that T cell depletion in gp91^-/-^ mice does not reduce the lesion size or tissue damage following VACV infection, suggesting that production of ROS by Ly6C^+^Ly6G^+^ cells modulates tissue damage in a T cell-independent manner. The exact mechanisms responsible for the profound damage found in the ear of mice infected with VACV remains unknown, and is a focus of our ongoing studies. The late time point at which damage occurs may reflect a role for antibody-mediated mechanisms that act through innate effector cells to initiate damage. Ly6C^+^Ly6G^+^ cells express several other molecules capable of suppressing immune responses, including CD163 and CD200. CD200-mediated suppression targets any cell expressing CD200R, including monocytes, macrophages, granulocytes, and T cells [43]. The activity of CD163, a scavenger receptor involved in the clearance of hemoglobin, leads to the up-regulation of the enzyme HO-1 [44]. In turn, this enzyme is both anti-inflammatory [45] and tissue-protective [46], [47], [48] through pathways involving CO, bilirubin, and Fe^2+^. In addition, ROS are critical mediators of signaling by cytokine and hormone receptors that may be required for tissue repair, such as insulin, platelet-derived growth factor, fibroblast growth factor and angiotensin [49]. Thus, Ly6C^+^Ly6G^+^ cells possess many mechanisms capable of modulating the immune response in order to provide tissue protection and repair.

The role of the Ly6C^+^Ly6G^-^ monocyte population in immunity to a peripheral virus infection is less defined, as there is currently no method available to specifically deplete these cells without affecting the Ly6C^+^Ly6G^+^ cells. Ly6C^+^Ly6G^-^ monocyte recruitment occurs prior to the recruitment of αβ T cells and coincides with the time point at which virus replication is controlled. By subtractive reasoning we are able to gain an insight into the role of these cells. The Ly6C^+^Ly6G^-^ monocyte population may be required for control of virus replication at the site of infection, but systemic depletion of populations including these cells does not substantially increase virus titers in the ear. Clearly cells that are depleted by treatment with clodronate liposomes do prevent systemic spread of the virus via an unknown mechanism that does not involve iNOS. Subcapsular sinus macrophages have been proposed as a gatekeeper cell type that prevents systemic spread of viruses [50], [51] and these cells are known to be infected following VACV infection [52]. However we observed no depletion of subcapsular sinus macrophages following systemic depletion with clodronate liposomes, ruling out these cells as the ones responsible for controlling systemic spread of VACV following intradermal infection. It is possible that clodronate-mediated depletion of Ly6C^+^Ly6G^-^ monocytes (or other cells) at sites other than the ear pinnae or subcapsular sinus is responsible for allowing systemic spread of VACV following peripheral infection, but we have been unable to identify specific populations of cells that are depleted by systemic administration of clodronate and definitely prevent virus spread.

The derivation of the Ly6C^+^Ly6G^+^ cell population we have described remains unknown. Ly6C^+^Ly6G^+^ cells are recruited from the blood at a time point after infection that is not normally associated with neutrophil recruitment. Ly6C^+^Ly6G^+^ cells have CD115 promoter activity at some point during their differentiation and display a mononuclear morphology but do not express the monocyte marker CX_3_CR1. The latter observation could be related to infection with VACV, as this virus expresses an immune modulator that causes the production of glucocorticoids [53], and in vivo administration of glucocorticoids can induce production of a monocytic cell type that downregulates expression of CX_3_CR1 [54]. These glucocorticoid-induced cells express many of the surface markers of myeloid derived suppressor cells (MDSC), a heterogeneous cell population described as suppressors of T cell responses in a tumor microenvironment [55], [56]. The Ly6C^+^Ly6G^+^ cells found at the site of VACV infection share expression of many surface markers with granulocytic MDSC, and produce large quantities of ROS, which MDSC use to modulate T cell activity [57], [58], [59]. However, MDSC are typically identified by expression of CD1b and Gr-1, a phenotype shared with neutrophils and inflammatory macrophages, and a full pathway of differentiation of these cells during resting or pathological conditions is yet to be published. The Ly6C^+^Ly6G^+^ cells we describe in this study share expression of some surface markers and some functions with neutrophil and monocyte populations, as well with MDSC. Without the definition of a widely agreed upon panel of markers that identify MDSC it is therefore impossible to conclude that the Ly6C^+^Ly6G^+^ cells are MDSC. Indeed, in the absence of definitive evidence that the Ly6C^+^Ly6G^+^ cells derive from a discrete lineage, their immunomodulatory function thus appears insufficient at this time to define the existence of a novel cell population.

In summary, we have identified and described the role of a distinct population of Ly6C^+^Ly6G^+^ cells that adds to the complexity of the phagocyte compartment [60]. These Ly6C^+^Ly6G^+^ cells are innate immune cells that regulate the destructive action of the innate immune system, reducing tissue damage and allowing wound healing. Modulation of the activity of these cells presents an attractive therapeutic strategy for preventing tissue damage in a wide range of infections and other pathologies.

## Materials and Methods

### Mice

All mice were housed in the specific pathogen free animal facility of the Hershey Medical Center. C57BL/6 mice were purchased from Charles River Laboratories (National Cancer Institute, Frederick, MD). iNOS^-/-^ mice [61] were purchased from Taconic. Gp91^-/-^ mice [33] and MAFIA mice [21] were purchased from Jackson Laboratory. All transgenic or knockout mouse strains were on the C57BL/6 background after a minimum of 12 backcrosses to this strain. Mouse strains, with the exception of C57BL/6, were subsequently bred at the Hershey Medical Center. All animals were maintained in microisolator cages and treated in accordance with the National Institutes of Health and American Association of Laboratory Animal Care (AAALAC International) regulations. All animal-related experiments and procedures were approved by the Penn State Hershey Institutional Animal Care and Use Committee.

### Virus

Mice were injected in each ear pinna with 10^4^ pfu VACV strain Western Reserve in a volume of 10 µl [8]. To analyze the presence of replicating virus, the ear pinnae or ovaries were harvested, subjected to three freeze/thaw cycles, homogenized and sonicated. Lysate was then placed on a monolayer of 143B cells, and a plaque assay was used to determine viral titer [8].

### Cell depletion

To deplete phagocytes, 200 µl of clodronate liposomes in PBS were injected i.v. on days 0, 1, 3, and 4 of infection [20]. This injection scheme effectively depletes monocytes and many macrophage populations [27], [62]. Cl_2_MDP (or clodronate) was a gift of Roche Diagnostics GmbH, Mannheim, Germany. Liposomes were prepared using Phosphatidylcholine (LIPOID E PC, Lipoid GmbH) and cholesterol (Sigma). Depletion of GFP^+^ cells in MAFIA mice was accomplished using AP20187 (Ariad Pharmaceuticals), as previously described [21]. AP20187 was diluted to a working concentration of 0.55 mg/ml in sterile water containing 4% ethanol, 10% PEG-400, and 1.7% Tween immediately before injection. AP20187 (10mg/kg) was injected i.v. daily for 5 days. Depletion was maintained with injections AP20187 (1mg/kg) every 3 days. Antibody-mediated depletion of Ly6G^+^ cells was by injection of 0.5 mg of anti-Ly6G (1A8, BioXCell) i.p. every 4 days [25].

### 5C6 (Anti-CD11b)-mediated blocking of phagocyte extravasation

Monocyte extravasation was partially blocked using the anti-CD11b antibody clone 5C6 [63]. Mice were injected i.v. with 0.5 mg/mouse of 5C6 antibody or an isotype control (rat IgG2b) every 24 hours. The clone 5C6 recognizes a epitope distinct from the anti-CD11b antibody clone M1/70 [63], so there is no 5C6-mediated interference in the detection of CD11b^+^ cells by flow cytometry.

### Analysis of the immune cell infiltrate

Immune cells were isolated as previously described [9], [10]. Briefly, ear pinnae were microdissected to increase surface area and incubated in 1 mg/ml collagenase XI (Sigma) for 30 min at 37°C. The tissue was then passed through metal screens to create a single cell suspension. RBC were lysed using ACK lysis buffer (Invitrogen). Cells were then incubated in Fc Block (BD) and stained in a solution of antibodies diluted in Fc Block and 10% mouse serum (Sigma). To detect intracellular markers or cytokines, cells were first fixed in 1% paraformaldehyde and stained and washed in the presence of 0.5% saponin (Sigma). Antibodies were from eBioscience unless noted otherwise and included anti-CD11b (M1/70), -CD11c (N418), -CD19 (eBio1D3), -CD68 (FA-11), -CD90 (53-2.1), -F4/80 (BM8), -IFN-α (RMMA-1), -IFN-β (RMMB-1), -iNOS (6), -Ly6C (AL-21), -Ly6G (1A8), -NK1.1 (PK136), -TCRβ (H57-597, BD Bioscience), -CD163 (ED2, Serotec), and -TNF-α (MP6-XT22). Streptavidin (BD Bioscience) was used to label biotin-conjugated antibodies. For detection of production of IFN-α, IFN-β and TNF-α, ex vivo cells were incubated in the presence of 5 µg/ml brefeldin A (Sigma) for 4 hours prior to staining. To detect TNF-α production, cells were incubated in the presence of 20 µg/ml CpG (Invivogen) for 2 hours prior to the addition of brefeldin A. For flow cytometry analysis all sample acquisition was with a FACsCanto or LSRII (BDBiosciences) in the Hershey Medical Center Flow Cytometry Core Facility. Data were analyzed using FlowJo software (Treestar) as outlined in Fig. S3.

### Reactive oxygen detection

Cells isolated from infected ear pinnae, as described above, were incubated in 20 mM CM-H_2_DCFDA in PBS for 30 min at 37°C and developed for an addition 4–6 hours in the dark. Fluorescence was detected by flow cytometry.

### Monocyte tracking

Tracking of classical monocytes was accomplished using fluorosphere labeling as previously described [27]. A 2.5% solids solution of 0.5μm FITC-labeled latex beads (Polysciences, Inc) was diluted 1∶25 in sterile PBS. A 250 µl dose of this dilution was injected i.v. alone or 1 day following an i.v. injection of 200 µl clodronate liposomes. Phagocytes were then isolated from ear pinnae, stained, and analyzed using flow cytometry as described above.

### Histology

Isolated cells were stained with a Phycoerythrin (PE)-conjugated anti-Ly6G antibody (clone 1A8), then incubated with anti-PE beads and separated using an AutoMACS sorter. The Ly6G^+^ and Ly6G^-^ fractions (which contained large amounts of debris that were disregarded) were analyzed using Romanowsky staining.

## Supporting Information

Figure S1 **Infiltration of CD11c^**+**^ to the site of peripheral VACV infection.** Wild-type mice were infected with 10^4^ pfu VACV in the ear pinnae and ears were harvested at the days shown. Cells were stained and analyzed as outlined in Fig. S3 below. CD11c^+^ cells represented a small fraction (around 15-20%) of infiltrating CD11b^+^ cells and expression of CD11c did not correlate with expression of other cellular markers (except CD11b) or functional phenotypes.
(TIF)

Figure S2 **Depletion of T cells does not lead to increased viral titer.** Wild-type mice were treated with either T24 (anti-Thy1) antibody (black bar) or isotype control (open bar) and infected i.d. in the ear pinnae with WR VACV. Ear pinnae and spleen were harvested at 7 days post-infection and assayed for both the presence of Thy1^+^ T cells in spleen and ear by flow cytometry, as well as for the presence of virus by plaque assay.
(TIF)

Figure S3 **Gating strategy.** For all analyses, cells were gated on a discernable scatter population that contained all CD11b^+^ cells and subsequently upon cells that could be definitively identified as singlets via the relationship of scatter area vs height or width. In some experiments cells staining positively for a “dump” gate of CD19 (B cells), CD90 (Thy1, T cells) and NK1.1 (NK cells) were discarded from the analysis. Of the resulting cells greater than half (on d5 post infection) typically expressed GFP in MAFIA mice and CD11b. Data shown are representative, and are from MAFIA mice 5d post infection with VACV.
(TIF)

Figure S4 **Both Ly6C^**+**^Ly6G^-^ and Ly6C^**+**^Ly6G^**+**^ subpopulations respond to i.d. infections with viruses other than VACV.** MAFIA mice were infected i.d. in the ear pinnae with either Herpes simplex virus (A) or adenovirus (B). Ear pinnae were harvested 5 days post-infection and the CD11b^+^ GFP/CD115^+^ monocyte population was then analyzed for Ly6C and Ly6G expression by flow cytometry.
(TIF)

Figure S5 **Inducible nitric oxide activity does not affect viral titer at the site of infection.** Wild-type (open squares) or Nos2-/- (filled circles) mice were infected i.d. in the ear pinnae with WR VACV. Ear pinnae were harvested at various days post-infection and lysates were used in a plaque assay.
(TIF)

Figure S6 **Isotype control antibody to anti-Ly6G does not increase tissue damage following VACV infection.** Mice were infected with VACV in the ear pinnae and treated with PBS (closed circles) or isotype control (open circles) for 1A8 anti-Ly-6G antibody (rat IgG2a) at day minus 1 and every 4 days subsequently. Tissue loss (hole size in the ear) was measured daily. Error bars represent S.E.M. from 10 ears. Similar to the result shown, no significant differences were observed between lesion size in mice treated with diluent or isotype control antibody.
(TIF)

Figure S7 **Infiltration of phagocytic cells is similar in wild-type and gp91^-/-^ mice.** Wild-type (filled diamonds) or gp91^-/-^ mice (open circles) were infected with VACV in the ear pinnae. Ears were harvested, digested to produce single cell suspensions and analyzed for the presence of CD11b^+^ cells (A) or CD11b^+^Ly6C^+^Ly6G^+^ cells (B). Similar total numbers of cells were obtained from each variety of mice, and numbers shown are percentages of total cells analyzed. Error bars represent S.E.M. from 4 ears.
(TIF)

Figure S8 **Ear swelling and tissue loss is similar in gp91^-/-^ mice treated with depleting anti-Ly6G antibody or isotype control.** Gp91^-/-^ mice were injected with 1A8 anti-Ly6G (open circles) or isotype control antibody (closed squares) 24 hr prior to infection with VACV in the ear pinnae and every 4 days subsequently for the duration of the experiment. Tissue swelling (A) and tissue damage (B) was measured at the times shown post infection. Error bars represent S.E.M. from 10 ears.
(TIF)
