# Supplementary figures and images for: CD11b+, Ly6G+ Cells Produce Type I Interferon and Exhibit Tissue Protective Properties Following Peripheral Virus Infection
Source: PLoS Pathog. 2011 Nov 10;7(11):e1002374. doi: 10.1371/journal.ppat.1002374 (PMC3213107; doi:10.1371/journal.ppat.1002374)

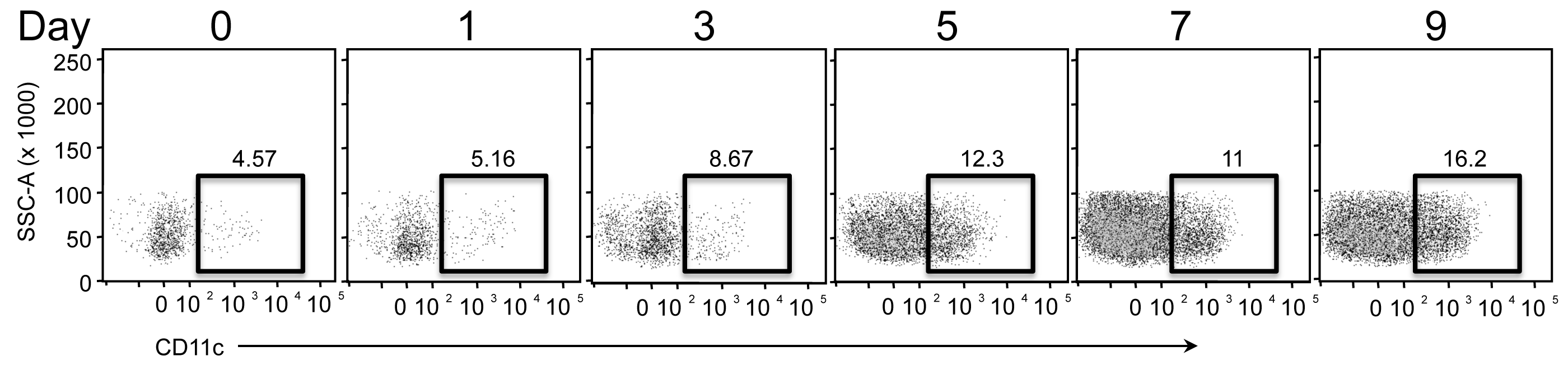

Supplement: Figure S1 — Infiltration of CD11c+ to the site of peripheral VACV infection. Wild-type mice were infected with 104 pfu VACV in the ear pinnae and ears were harvested at the days shown. Cells were stained and analyzed as outlined in Fig. S3 below. CD11c+ cells represented a small fraction (around 15-20%) of infiltrating CD11b+ cells and expression of CD11c did not correlate with expression of other cellular markers (except CD11b) or functional phenotypes. (TIF) [file ppat.1002374.s001.tif]

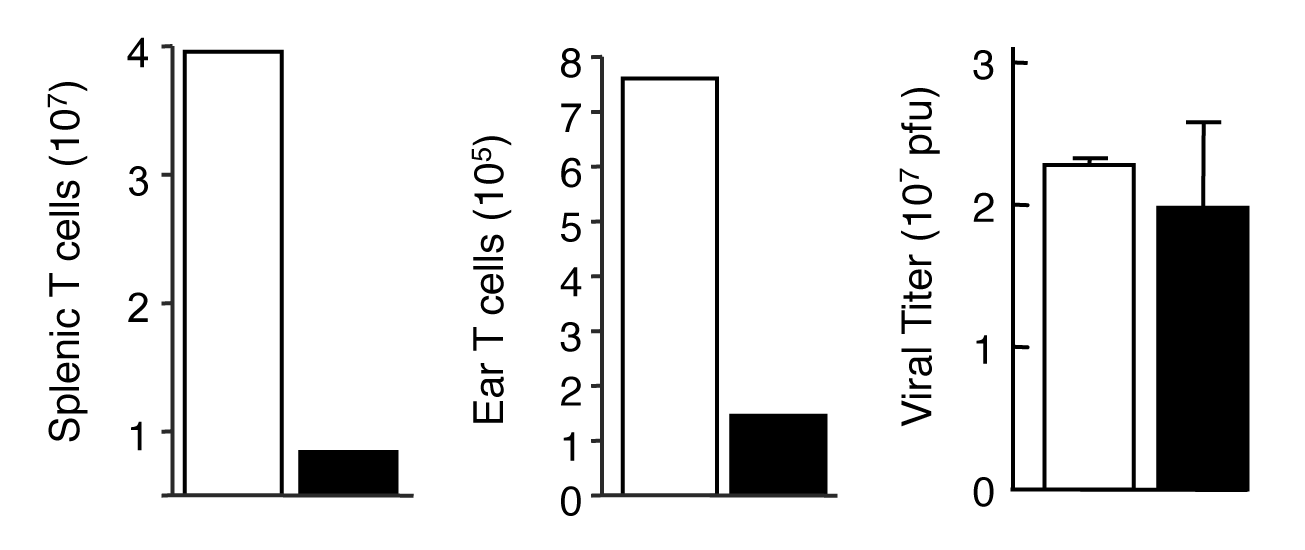

Supplement: Figure S2 — Depletion of T cells does not lead to increased viral titer. Wild-type mice were treated with either T24 (anti-Thy1) antibody (black bar) or isotype control (open bar) and infected i.d. in the ear pinnae with WR VACV. Ear pinnae and spleen were harvested at 7 days post-infection and assayed for both the presence of Thy1+ T cells in spleen and ear by flow cytometry, as well as for the presence of virus by plaque assay. (TIF) [file ppat.1002374.s002.tif]

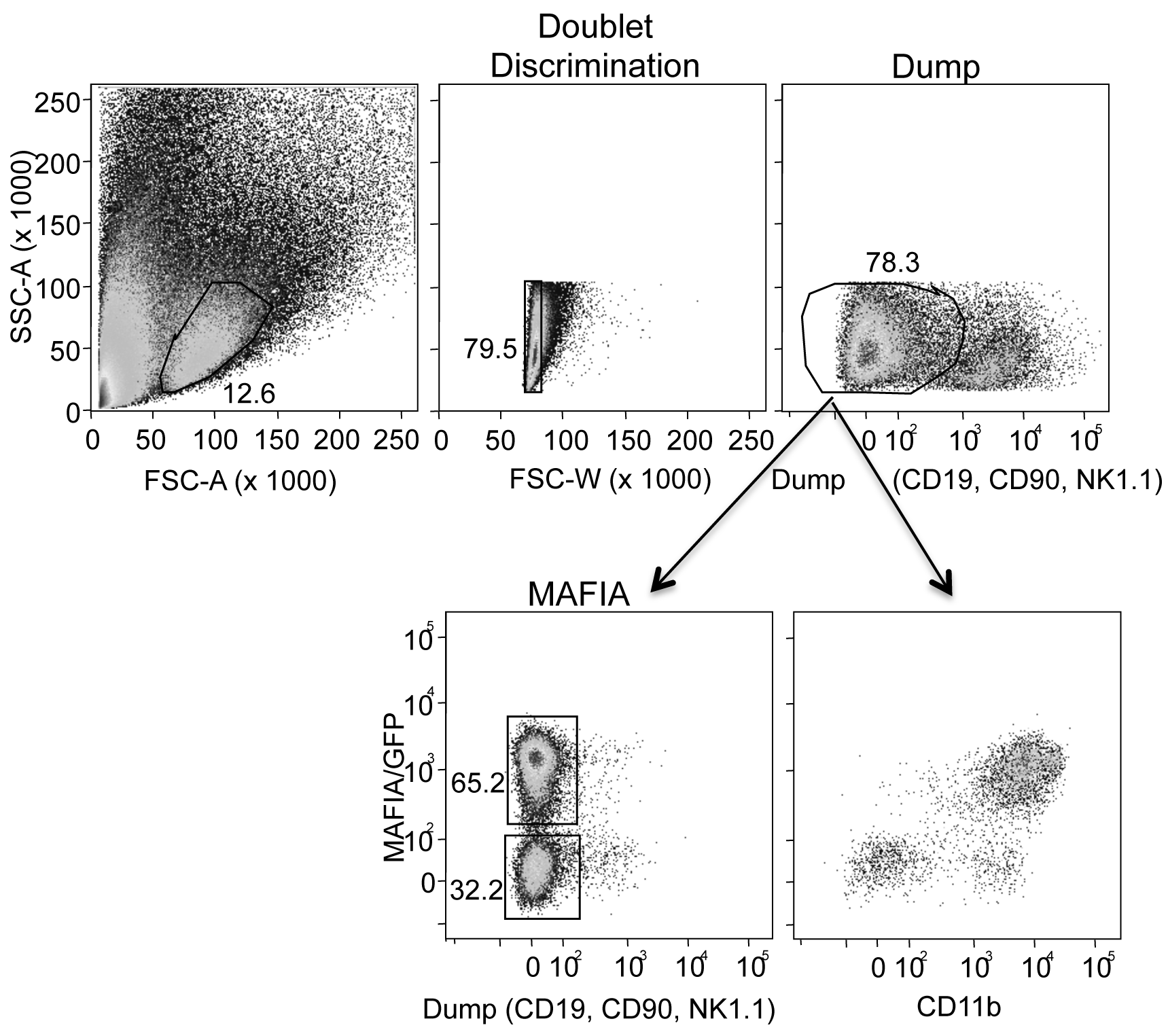

Supplement: Figure S3 — Gating strategy. For all analyses, cells were gated on a discernable scatter population that contained all CD11b+ cells and subsequently upon cells that could be definitively identified as singlets via the relationship of scatter area vs height or width. In some experiments cells staining positively for a “dump” gate of CD19 (B cells), CD90 (Thy1, T cells) and NK1.1 (NK cells) were discarded from the analysis. Of the resulting cells greater than half (on d5 post infection) typically expressed GFP in MAFIA mice and CD11b. Data shown are representative, and are from MAFIA mice 5d post infection with VACV. (TIF) [file ppat.1002374.s003.tif]

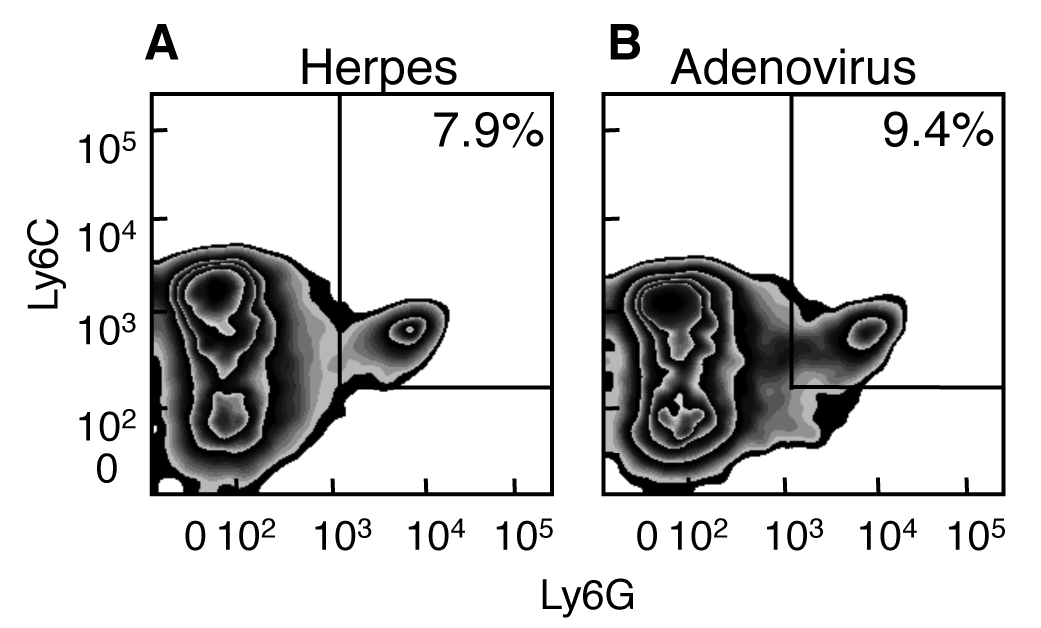

Supplement: Figure S4 — Both Ly6C+Ly6G- and Ly6C+Ly6G+ subpopulations respond to i.d. infections with viruses other than VACV. MAFIA mice were infected i.d. in the ear pinnae with either Herpes simplex virus (A) or adenovirus (B). Ear pinnae were harvested 5 days post-infection and the CD11b+ GFP/CD115+ monocyte population was then analyzed for Ly6C and Ly6G expression by flow cytometry. (TIF) [file ppat.1002374.s004.tif]

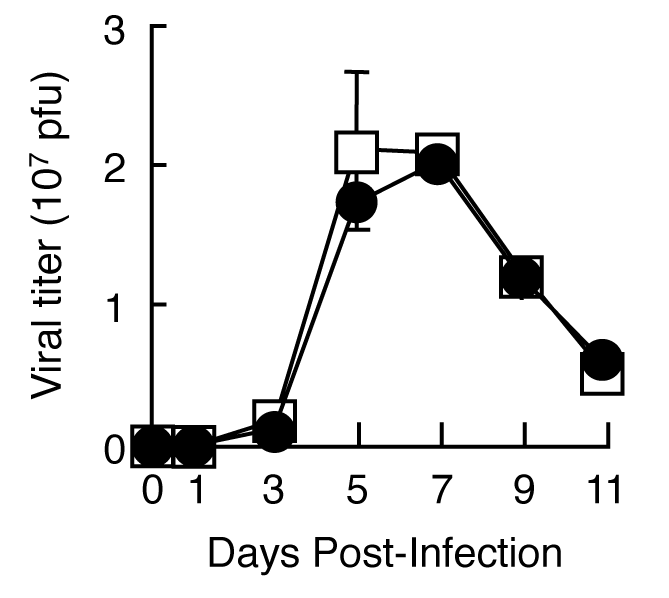

Supplement: Figure S5 — Inducible nitric oxide activity does not affect viral titer at the site of infection. Wild-type (open squares) or Nos2-/- (filled circles) mice were infected i.d. in the ear pinnae with WR VACV. Ear pinnae were harvested at various days post-infection and lysates were used in a plaque assay. (TIF) [file ppat.1002374.s005.tif]

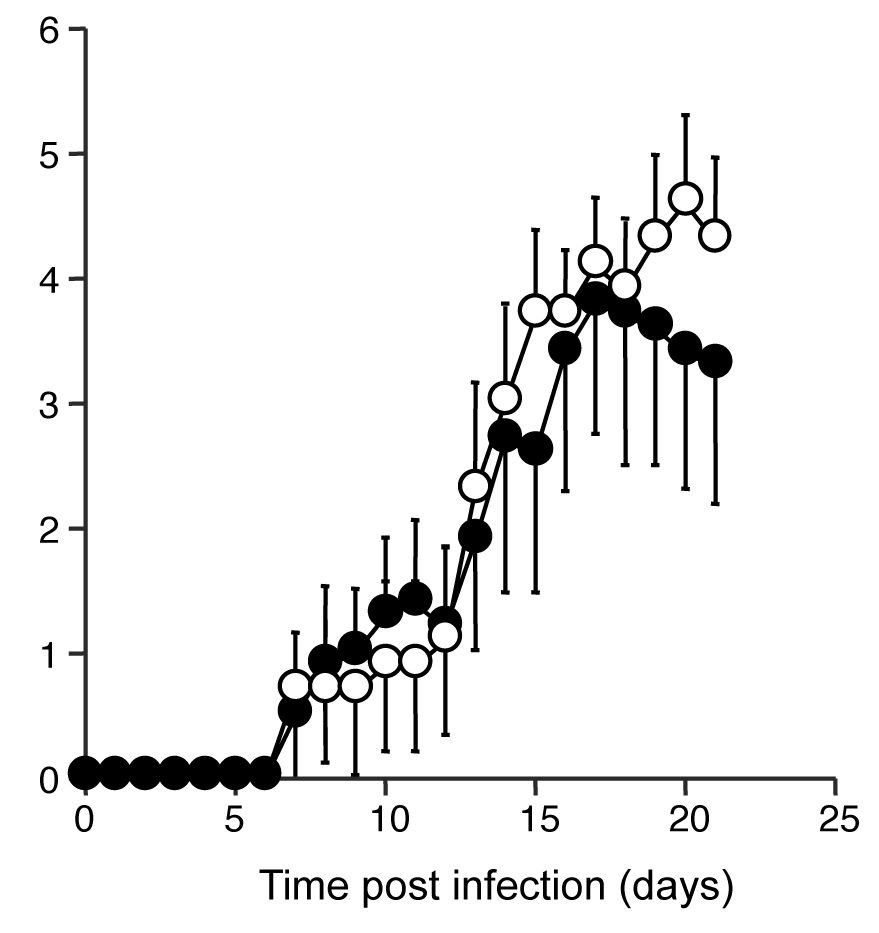

Supplement: Figure S6 — Isotype control antibody to anti-Ly6G does not increase tissue damage following VACV infection. Mice were infected with VACV in the ear pinnae and treated with PBS (closed circles) or isotype control (open circles) for 1A8 anti-Ly-6G antibody (rat IgG2a) at day minus 1 and every 4 days subsequently. Tissue loss (hole size in the ear) was measured daily. Error bars represent S.E.M. from 10 ears. Similar to the result shown, no significant differences were observed between lesion size in mice treated with diluent or isotype control antibody. (TIF) [file ppat.1002374.s006.tif]

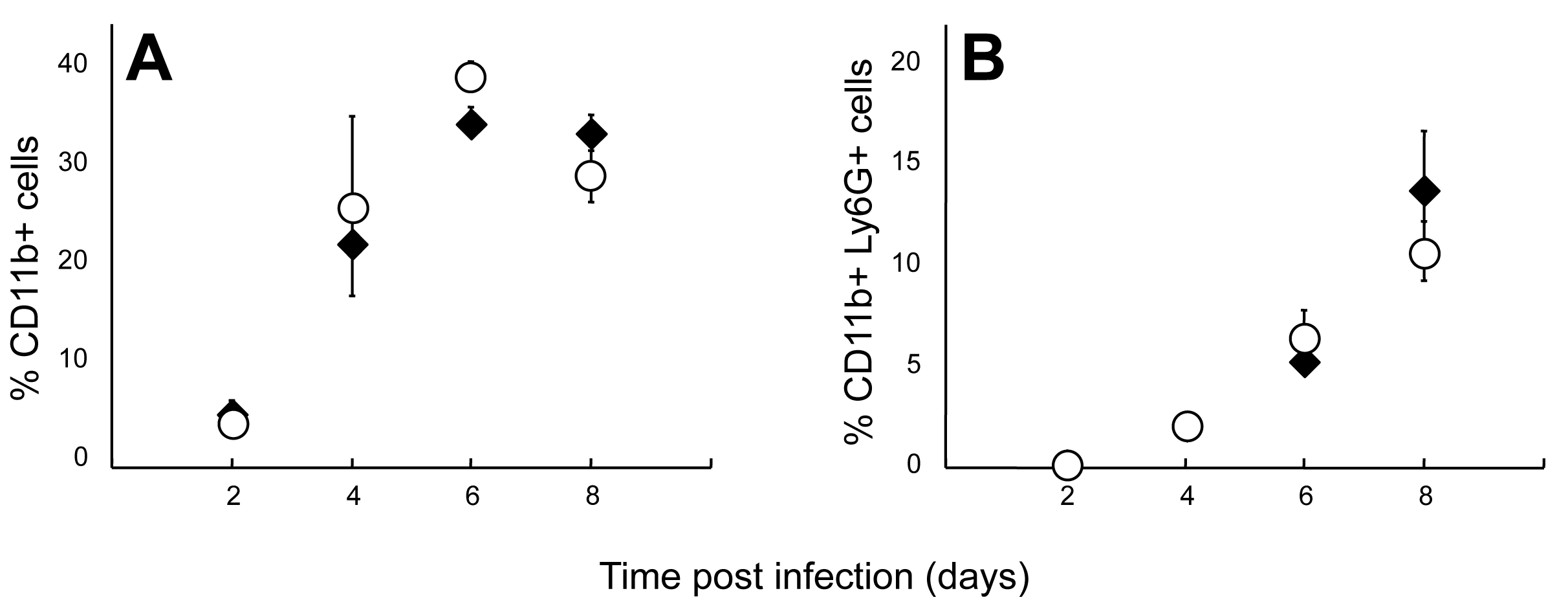

Supplement: Figure S7 — Infiltration of phagocytic cells is similar in wild-type and gp91-/- mice. Wild-type (filled diamonds) or gp91-/- mice (open circles) were infected with VACV in the ear pinnae. Ears were harvested, digested to produce single cell suspensions and analyzed for the presence of CD11b+ cells (A) or CD11b+Ly6C+Ly6G+ cells (B). Similar total numbers of cells were obtained from each variety of mice, and numbers shown are percentages of total cells analyzed. Error bars represent S.E.M. from 4 ears. (TIF) [file ppat.1002374.s007.tif]

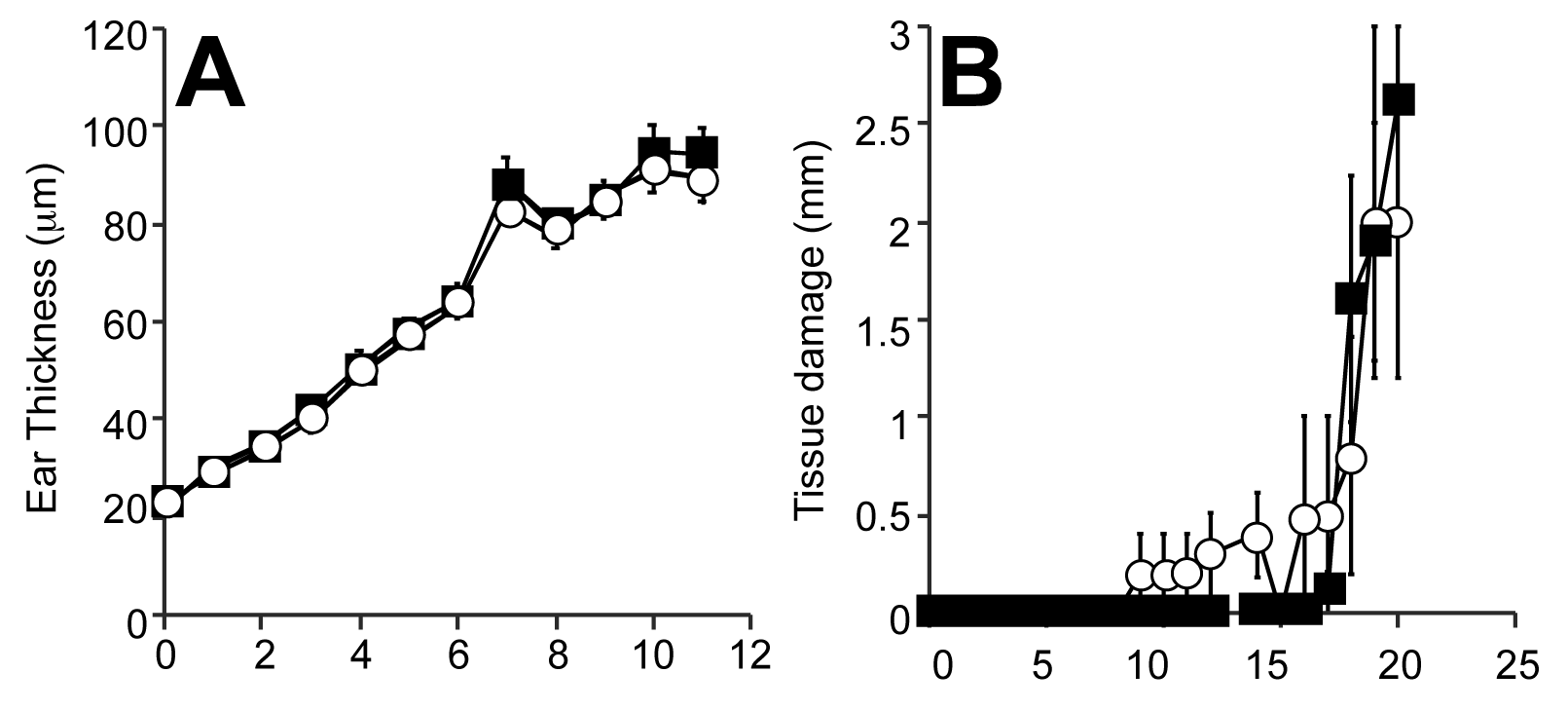

Supplement: Figure S8 — Ear swelling and tissue loss is similar in gp91-/- mice treated with depleting anti-Ly6G antibody or isotype control. Gp91-/- mice were injected with 1A8 anti-Ly6G (open circles) or isotype control antibody (closed squares) 24 hr prior to infection with VACV in the ear pinnae and every 4 days subsequently for the duration of the experiment. Tissue swelling (A) and tissue damage (B) was measured at the times shown post infection. Error bars represent S.E.M. from 10 ears. (TIF) [file ppat.1002374.s008.tif]
